# Effects of exercise training with intermittent hyperoxic intervention on endurance performance and muscle metabolic properties in male mice

**DOI:** 10.14814/phy2.16117

**Published:** 2024-06-19

**Authors:** Junichi Suzuki

**Affiliations:** ^1^ Laboratory of Exercise Physiology, Health and Sports Sciences, Course of Sports Education, Department of Education Hokkaido University of Education Iwamizawa Hokkaido Japan

**Keywords:** Bayesian data analysis, endurance exercise, fatty acid metabolism, intermittent hyperoxia, nuclear N‐terminal PGC1alpha

## Abstract

This study aimed to investigate how intermittent hyperoxic exposure (three cycles of 21% O_2_ [10 min] and 30% O_2_ [15 min]) affects exercise performance in mice. Three hours after the acute exposure, there was an observed increase in mRNA levels of phosphofructokinase (Bayes factor [BF] ≥ 10), mitochondrial transcription factor‐A (BF ≥10), PPAR‐α (BF ≥3), and PPAR‐γ (BF ≥3) in the red gastrocnemius muscle (Gr). Four weeks of exercise training under intermittent (INT), but not continuous (HYP), hyperoxia significantly (BF ≥30) increased maximal exercise capacity compared to normoxic exercise‐trained (ET) group. INT group exhibited significantly higher activity levels of 3‐hydroxyacyl‐CoA‐dehydrogenase (HAD) in Gr (BF = 7.9) compared to ET group. Pyruvate dehydrogenase complex activity levels were significantly higher in INT group compared to ET group in white gastrocnemius, diaphragm, and left ventricle (BF ≥3). NT‐PGC1α protein levels in Gr (BF = 7.7) and HAD activity levels in Gr (BF = 6.9) and soleus muscles (BF = 3.3) showed a significant positive correlation with maximal work values. These findings suggest that exercise training under intermittent hyperoxia is a beneficial strategy for enhancing endurance performance by improving fatty acid and pyruvic acid utilization.

## 1 INTRODUCTION

Since the 1990s, exercise training under a hypoxic environment has been developed to enhance athletic performance at sea level (Terrados et al., 1990; Vogt & Hoppeler, 2010). More recently, hyperoxic training, which involves exercise training under hyperoxic environment, has gained popularity among athletes and has been extensively studied to understand the mechanisms behind its performance‐enhancing effects. Cycling exercise under hyperoxia with 50% oxygen has been shown to improve maximal power output and endurance capacity in humans (Ulrich et al., 2017). Similarly, exercising under hyperoxia with 70% oxygen at the same absolute workload as normoxia showed lower levels of ventilation, heart rate, blood lactate, and catecholamines (Byrnes et al., 1984). While maximal oxygen consumption values were similar between normoxia and hyperoxia (100% O_2_) in untrained individuals, after 8 weeks of endurance training, the values were significantly higher (1.4‐fold) in the hyperoxic group (Broxterman et al., 2023). These findings suggest that daily exercise training under hyperoxia or intermittent hyperoxia may be less physically demanding procedure, particularly for endurance‐trained individuals.

Cellular responses to hypoxia are well‐documented. Under hypoxic conditions, the α‐subunit of hypoxia‐inducible factor‐1 (HIF1α) becomes stable and moves into the nucleus. This leads to the activation of HIF1α‐responsive genes (Ivan et al., 2001; Maxwell et al., 1999). Once HIF1α is stabilized for a certain period, PHD2, its target, breaks it down in a negative feedback mechanism (Bruick & McKnight, 2001), primarily within the nucleus of the cell (Pientka et al., 2012).

Recently, there has been a proposal suggesting that changes in oxygen availability, rather than consistent hypoxic or hyperoxic conditions, have a significant impact on HIF transcriptional effects. In a study conducted on humans, it was observed that breathing 100% oxygen for 2 h, followed by 36 h of breathing room air, led to a 60% increase in serum erythropoietin levels (Balestra et al., 2006). In cultured human umbilical vein endothelial cells, there was no signal of HIF1α protein immediately after exposure to hyperoxia (32% O_2_ for 2 h), but after 4 and 6 h of recovery under 21% O_2_, there was a significant upregulation of HIF1α protein levels (Cimino et al., 2012). Therefore, it is likely that hyperoxic exposure followed by normoxia is interpreted as a hypoxic event at the cellular level, and this is referred to as the hyperoxic‐hypoxic paradox (Balestra et al., 2006; Cimino et al., 2012; Salvagno et al., 2023).

Consequently, it is postulated that the combined effects of hypoxia on exercise performance are comparable to those of hyperoxia. Recent research has demonstrated that exercise training under intermittent hypoxia (three cycles of 21% [10 min] and 14% O_2_ [15 min]), as opposed to continuous hypoxia, has a positive effect on improving endurance performance. This improvement is accomplished through the enhancement of citrate synthase (CS) and cytochrome oxidase (COX) activity levels, as well as the elevation of nuclear N‐terminal (NT) isoform of PGC1α protein levels in hind–leg muscles (Suzuki, 2022).

If exercising under intermittent hyperoxia yields similar results, it could potentially be a beneficial strategy for enhancing endurance performance. As mentioned earlier, exercising under hyperoxia has been shown to alleviate physical stress, making it suitable for conducting rigorous training programs over extended periods. However, none of the previous studies have examined the profiles of intermittent hyperoxia on muscle metabolic properties and exercise performance.

To confirm this idea, the present study aimed to determine whether acute intermittent hyperoxia itself, that is, without acute exercise‐induced response, promotes the expression levels of HIF‐target genes and proteins as well as genes that mediate muscle metabolism. The second objective of this study was to investigate how chronic exercise training with intermittent hyperoxic intervention improves endurance capacity and muscle metabolic properties. The NT‐PGC1α has been identified as playing a regulatory role in mitochondrial biogenesis and promoting the adaptation of muscle metabolism induced by endurance exercise (Wen et al., 2014). Therefore, measuring NT‐PGC1α levels may provide insight into potential modes of adaptation induced by exercise under intermittent hyperoxia.

## 2 MATERIALS AND METHODS

### 2.1 Ethical approval

All procedures were approved by the Animal Care and Use Committee of Hokkaido University of Education (No. 7, approved on 2023/4/1) and conducted in accordance with the “Guiding Principles for the Care and Use of Animals in the Field of Physiological Sciences” of the Physiological Society of Japan.

### 2.2 Animals

Male MCH(ICR)/jcl mice, aged 10 weeks, were purchased from Clea Japan (Tokyo, Japan). They were housed under controlled conditions, with a temperature of 24 ± 1°C and a relative humidity of approximately 50%. Lighting was automatically controlled from 7:00 to 19:00. Each mouse was housed individually in a cage and provided with commercial laboratory chow (solid CE‐2; Clea Japan) and tap water ad libitum. After a 2‐week acclimation period, the mice were assigned to each experiment.

### 2.3 Experiment 1: Acute responses to intermittent hyperoxic exposure

The mice were randomly assigned to three groups: a normoxic control group (Cnt, *n* = 5), a hyperoxic exposure group (Hyp, exposed to 30% O_2_ for 75 min, *n* = 5), and an intermittent hyperoxic exposure group (Int, exposed to three cycles of 30% O_2_ for 15 min and room air for 10 min, *n* = 5). The author chose a small sample size for Experiment 1 because the effects of acute intermittent hyperoxic exposure were being evaluated for the first time. The initial intention was to gather basic evidence regarding the use of this protocol in chronic experimental designs. In this reason, red and white regions of gastrocnemius muscle were used to determine acute responses in representative of highly oxidative and highly glycolytic muscle regions.

Hyperoxic exposure was achieved by inflating a mixture of room air and oxygen gas (100%) into a chamber (length 0.48 m, width 0.35 m, and height 0.15 m) to achieve normobaric hyperoxia (30% O_2_). The oxygen concentration was monitored using an oxygen sensor (GOX‐100; Greisinger, Germany). The air in the chamber was circulated through a CO_2_ absorbent (Litholyme; Allied Healthcare Products, St. Louis, MO, USA) to maintain a CO_2_ concentration below 1000 ppm. When the O_2_ concentration was reduced from 30% to 21%, room air was inflated into the chamber. The order of each intervention was randomized, and mice were randomly assigned to each group.

Tissues were collected 3 h after each treatment. The mice were anesthetized with 3% sevoflurane (193–17,791; Fujifilm‐Wako, Osaka, Japan) inhalation, and the adequacy of anesthesia was validated using a toe pinch response. The gastrocnemius muscle was excised, and the deep red region (Gr) of the gastrocnemius was isolated from the superficial white region (Gw). The mice were killed by excision of the heart. All tissue samples were frozen in liquid nitrogen and stored at −80°C until further analysis.

### 2.4 Experiment 2: Chronic response of exercise under intermittent hyperoxia

In Experiment 2, the chronic response of exercise under intermittent hyperoxia was investigated. Forty male mice (10 weeks old) were randomly assigned to either the sedentary control group (SED, *n* = 10) or the training group (*n* = 30). In order to familiarize the mice with the treadmill device, mice in the training group underwent treadmill walking three times a week during the second week of the acclimation period. This was done using a controlled treadmill (Modular motor assay, Columbus Instruments, Columbus, OH, USA) at a speed of 10–15 m min^−1^ for 3 min per day with a 5 (π/180) rad incline. Following the acclimation period, the mice in the training group underwent a maximal exercise capacity test using a graded ramp running protocol on the controlled treadmill, as previously reported (Suzuki, 2022). Total work (J, kg·m^2^·s^−2^) was calculated by multiplying body weight (kg), speed (m s^−2^), time (sec), slope (%), and 9.8 (m s^−2^). Exhaustion was defined as the mouse staying on the metal grid (no electrical shock was applied) at the rear of the treadmill for more than 5 s, despite external gentle touch being applied to their tail with a conventional elastic bamboo stick (0.8 mm in diameter). Following the performance test, the mice were given a 48‐h non‐exercise period before starting treadmill training.

**FIGURE 1:**
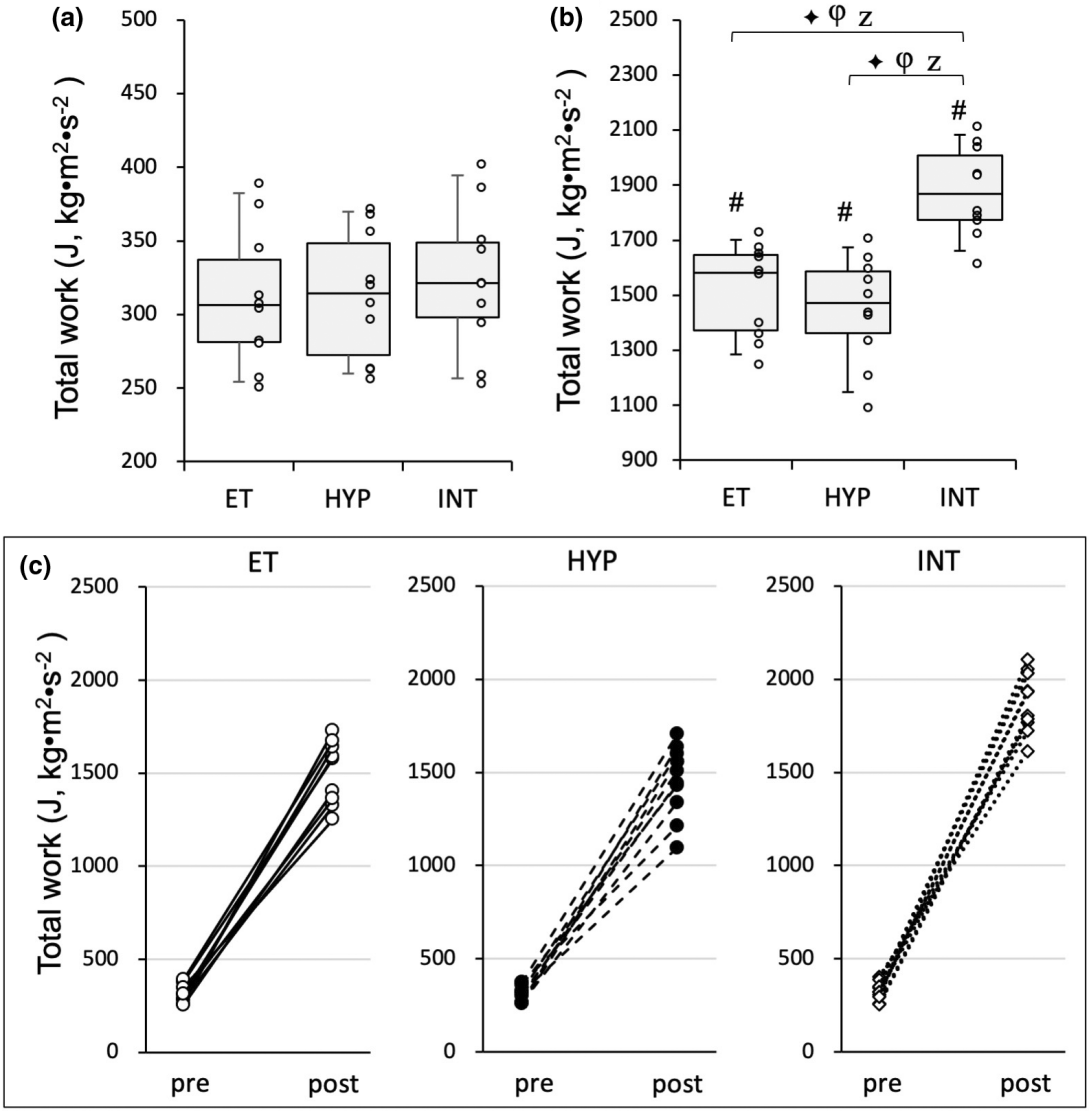
Endurance exercise performance test in the Experiment 2. Total work capacity of the endurance capacity test (a) before and (b) after 4 weeks of treadmill exercise training, and (c) individual changes in total work values before and after the training. Values are expressed as box and whisker plots with 5th, 25th, 50th, 75th, and 95th percentile. Dots are individual data points. #, significantly different from pre‐treadmill training values of each group shown in the panel (a). Bayes factor: ≥30. Bayes factor: ✦, ≥30. φ, the 95% confidential interval did not contain the mean value of target group for comparison. Z, as a reference value, NHST‐ANOVA and Tukey's post hoc test, *p* < 0.05.

Mice in the training group were divided into three groups: a normoxic exercise‐trained group (ET, *n* = 10), a hyperoxic exercise‐trained group (HYP, *n* = 10), and an exercise‐trained under intermittent hyperoxia group (INT, *n* = 10). This was done to ensure that the mean and standard deviation (SD) values of pre‐training results for total work were matched (Figure 1a). All mice in the trained groups successfully completed the exercise training, and their results were included in this study.

The mice in the training groups underwent endurance exercise training for 4 weeks, 6 days a week. The endurance exercise lasted for 75 min with a 10 (π/180) rad incline. Instead of using an electrical shock, the mice were motivated to run by touching their tail or planta pedis with a conventional test tube blush made of soft porcine bristles when they stayed on a metal grid for more than 3 s. The HYP and INT groups followed the same exercise protocols as the ET group for 3 days a week (Monday, Wednesday, and Friday). On the other days (Tuesday, Thursday, and Saturday), the HYP group exercised under hyperoxia (30% O_2_) and the INT group exercised under intermittent hyperoxia (three cycles of 30% O_2_ for 15 min and room air for 10 min). A treadmill (KN‐73, Natsume Co., Tokyo, Japan) was used for the daily exercise training. When the mice ran under hyperoxia, the runway of the treadmill was covered with a translucent plastic film to create a runway chamber (length 1.10 m, width 0.78 m, and height 0.3 m). To maintain the desired O_2_ concentration in the chamber, room air or 100% O_2_ was introduced into the chamber, and the O_2_ concentration was monitored as described above. To ensure that the CO_2_ concentration remained below 1000 ppm, the air in the chamber was circulated through a CO_2_ absorbent, as described above.

On the first day of training, the mice ran for 75 min at 18 m min^−1^ with a 10 (π/180) rad incline. The speed was gradually increased by 1 m min^−1^ every 3 days during the training period. Each exercise intervention took place between 5 and 9 am, and the order of the interventions was randomized daily. Forty‐eight hours after the final training session, the maximal exercise capacity of mice in the training groups was determined using the method described above.

Forty‐eight hours after the performance test, the mice were anesthetized as described in Experiment 1. The soleus (SOL), plantaris (PL), and gastrocnemius muscles were excised, and the deep red region (Gr) of the gastrocnemius was separated from the superficial white region (Gw). The diaphragm (DIA) was also excised. All samples were frozen in liquid nitrogen for biochemical analyses. The mice were killed by excision of the heart. After excision, the whole heart and left ventricle (LV) were weighed. All tissue samples were stored at −80°C until further analyses.

### 2.5 RNA isolation and cDNA synthesis

The mRNA‐containing fractions were isolated using RNAzol RT (RN190, Molecular Research Center, Ohio, USA). To determine mRNA, 4.5 μg of the mRNA‐containing fraction was used for cDNA synthesis. This was done by using an oligo dT primer (FSK‐201, Toyobo, Osaka, Japan) and Mmlv reverse transcriptase (TRT‐101, ReverTra Ace, RNase H minus point mutant, Toyobo).

### 2.6 Real‐time PCR analyses

mRNA expression levels were determined using a standard real‐time polymerase chain reaction (PCR) with the KAPA SYBR FAST qPCR Kit (KK4602, KAPA Biosystems, MA, USA). The sequences of the forward and reverse primer sets are shown in Table 1. All primer sets were purchased from Life technologies (Tokyo, Japan). Hypoxanthine ribosyltransferase (HPRT) were used as endogenous controls for mRNA expression analyses (Table 1). The PCR conditions for mRNA were as follows: 1 min pre‐denaturation at 95°C, and then 10 s denaturation at 95°C, 20 s annealing at 60, 60.5, or 63°C, and 1 s extension at 70°C for 40 cycles. A real‐time analysis of PCR amplification was performed on a CFX96 real‐time PCR system and analyzed with the CFX Manager software (Bio‐Rad, Hercules, CA, USA). Serial 5‐fold dilutions of a cDNA sample were used to generate a standard curve. Non‐specific products, such as primer dimer formation, were checked by dissociation curves and the results of negative control samples without cDNA.

**TABLE 1:** Sequences of forward and reverse primer sets used for PCR amplification of genes.

Target	Forward primer (5′ to 3′)	Forward primer (5′ to 3′)	GenBank accession no.
VEGFA	GCACTGGACCCTGGCTTTACTGCTG ID: H0184H11	ACGGCAATAGCTGCGCTGGTAGAC ID: H0184H12	NM_001025250.3
eNOS	TAGGGCTCGGGCTGGGTTTA ID: K4281A07	TACAGGGCCCATCCTGCTGA ID: K4281A08	NM_008713.4
PFK	CGGAGGAGAGCTAAAACTACAAGAG ID: K7648G01	CTTGGTAACCCTCATGGACAAAGA ID: K7648G02	NM_001163487.1
TFAM	TCCCCTCGTCTATCAGTCTTGTC ID: M1714G09	TCCACAGGGCTGCAATTTTCC ID: M1714G10	NM_009360.4
MEF2A	AGCGGAGACTCGGAATTGCAT ID: M5761G02	GGGCTGCCGTTGAAATTGTCT ID: M5761G03	NM_001291191.1
PPARα	TGCATTTGGGCGTATCTCACC ID: M2755H11	CAGAGCGCTAAGCTGTGATGA ID: M2755H12	NM_011144.6
PPARδ	GGGAAAAGTTTTGGCAGGAGC ID: K7882G06	CAGATGGACTGCCTTTACCGTG ID: K7882G07	NM_011145.3
PPARγ	GAGCCTGTGAGACCAACAGC ID: M5761G04	GTTGGTGGGCCAGAATGGCA ID: M5761G05	NM_001127330.2
HPRT (standard)	CGACCCTCAGTCCCAGCGTCGTGATTA ID: G0890B07	AGGGCCACAATGTGATGGCCTCCCA ID: G0890B08	NM_013556.2

Abbreviations: eNOS, endothelial nitric oxide synthase; HPRT, hypoxanthine ribosyltransferase; MEF2A, myocyte enhancer factor 2A, PPAR, peroxisome proliferator‐activated receptor; PFK, phosphofructokinase; TFAM, transcription factor A, mitochondrial; VEGFA, vascular endothelial growth factor‐A.

### 2.7 Sample preparation for biochemical analyses

A cytoplasmic or nuclear fraction of protein was obtained separately using precisely the same protocols as previously reported by the author (Suzuki, 2022). The efficacy of the separation was confirmed previously (Suzuki, 2022 [Figure S2]) by western blot using anti‐GAPDH antibody (a cytoplasmic marker, sc‐166574; Santa Cruz Biotechnology, Dallas, TE, USA) and anti‐Lamin A/C antibody (a nuclear marker, sc‐376,248; Santa Cruz).

Frozen tissue powder was obtained using a frozen sample crusher (SK mill; Tokken, Chiba, Japan) and homogenized with ice‐cold medium (10 mM HEPES buffer, pH 7.4; 1% NP‐40 [Fujifilm‐Wako]; 11.5% [w/v] sucrose; and 5% [v/v] protease inhibitor cocktail [P2714; Sigma‐Aldrich, St. Louis, MD, USA]) in an ultrasonic bath (43 kHz, 50 W) at 4°C for 5 min. It was then gently rotated at 4°C for 10 min. After centrifugation at 18,000 *g* and at 4°C for 10 min, the supernatant, cytoplasmic fraction, was collected and stored at −80°C. The pellet was resuspended with ice‐cold buffer described above without NP‐40 and rotated at 4°C for 10 min. After centrifugation as described above, the supernatant was drained, and the pellet was resuspended with ice‐cold buffer (10 mM HEPES buffer, pH 7.4; 1.5 mM MgCl_2_, 420 mM NaCl, 25% glycerol, and 5% [v/v] protease inhibitor cocktail). It was then rotated at 4°C for 10 min. After centrifugation at 18,000 g and at 4°C for 10 min, the supernatant, nuclear extraction, was used for western blot analysis. Total protein concentrations were measured using the Bradford assay (0.01% [w/v] CBB G‐250 [B3193, Tokyo Chemical Industry, Tokyo, Japan], 5% [v/v] ethanol [054‐07225, Fujifilm‐Wako, Osaka, Japan], 8.5% [v/v] phosphoric acid [164‐02176, Fujifilm‐Wako]) with bovine serum albumin (21011, iNtRON Biotechnology, Korea) as a standard.

### 2.8 Western blot analyses

A sample containing 30 μg of nuclear protein or 60 μg of cytoplasmic protein was heated at 99°C for 5 min with a Laemmli sample buffer (161‐0747, Bio‐Rad Laboratories, Hercules, CA, USA). Then, sample was separated on 12% polyacrylamide gels (TGX StainFree FastCast gel, 1610185, Bio‐Rad) using SDS/PAGE. The gels were exposed to ultra‐violet (UV) light for 1 min, and total protein patterns were visualized using the ChemiDoc MP Imager (Bio‐Rad). The stain‐free gel contains a trihalo compound that reacts with proteins during separation, making them detectable with UV exposure (Gilda & Gomes, 2013). The gels were then electrophoretically transferred to a polyvinylidene fluoride membrane. The blot was blocked with 5% non‐fat dry milk (sc‐2325; Santa Cruz Biotechnology, Dallas, TE, USA) in 0.1 M phosphate‐buffered saline (PBS) with 0.05% Tween20 for 1 h. Next, the blot was exposed to a specific primary antibody against NT‐PGC1α (1:1000, sc‐518,025, Santa Cruz), PHD2 (1:1000, sc‐271,835, Santa Cruz), fatty acid binding protein (FABP, 1:2000, sc‐514208, Santa Cruz), mitochondrial transcription factor A (TFAM, 1:1500, sc‐166965, Santa Cruz), mitofusion (MFN) 2 (1:1500, sc‐100560, Santa Cruz), and dynamin‐related protein (DRP) 1 (1:1500, sc‐271583, Santa Cruz) diluted in blocking buffer for 1 h. After incubating the blot with a HRP‐labeled mouse IgGκ light chain binding protein (1:5000, sc‐516102, Santa Cruz), it was reacted with Clarity Western ECL substrate (170‐5060, Bio‐Rad Laboratories), Clarity Max Western ECL substrate (1705062, Bio‐Rad Laboratories), or a mixture of both. The target proteins were detected with the ChemiDoc MP (Bio‐Rad Laboratories). The densities of the specific bands were quantified using Image Lab software (Bio‐Rad Laboratories) and normalized to the densities of all protein bands in each lane on the membrane (Suzuki, 2021). This normalizing procedure was confirmed to be superior to using β‐actin as a loading control (Gilda & Gomes, 2013). Subsequently, the normalized densities of the bands were further normalized to the same sample that was run on every gel and transferred to every membrane, as reported by the author (Suzuki, 2021).

### 2.9 Biochemical analyses of enzyme activity

The activity of 3‐hydroxyacyl‐CoA‐dehydrogenase (HAD) was assayed using the method described by Bass et al. (1969). The activity of citrate synthase (CS) was assayed following the methods of Srere (1969). Pyruvate dehydrogenase complex (PDHc) activity was assayed according to the method of Ke et al. (2014). The activity of carnitine palmitoyl transferase (CPT) 2 was assayed as previously reported by Suzuki (2021). Specific lactate dehydrogenase activities, pyruvate‐to‐lactate (LDH‐PL) or lactate‐to‐pyruvate (LDH‐LP) conversions, were determined following the protocol of Howell et al. (1979) with some modifications as reported previously (Suzuki, 2022). All measurements were carried out at 25°C using a spectrophotometer (U‐2001; Hitachi Co., Tokyo, Japan), and enzyme activities were reported as micromoles h^−1^ milligram of protein^−1^. Total protein concentrations were measured as described above.

### 2.10 Statistical analyses

**FIGURE 2:**
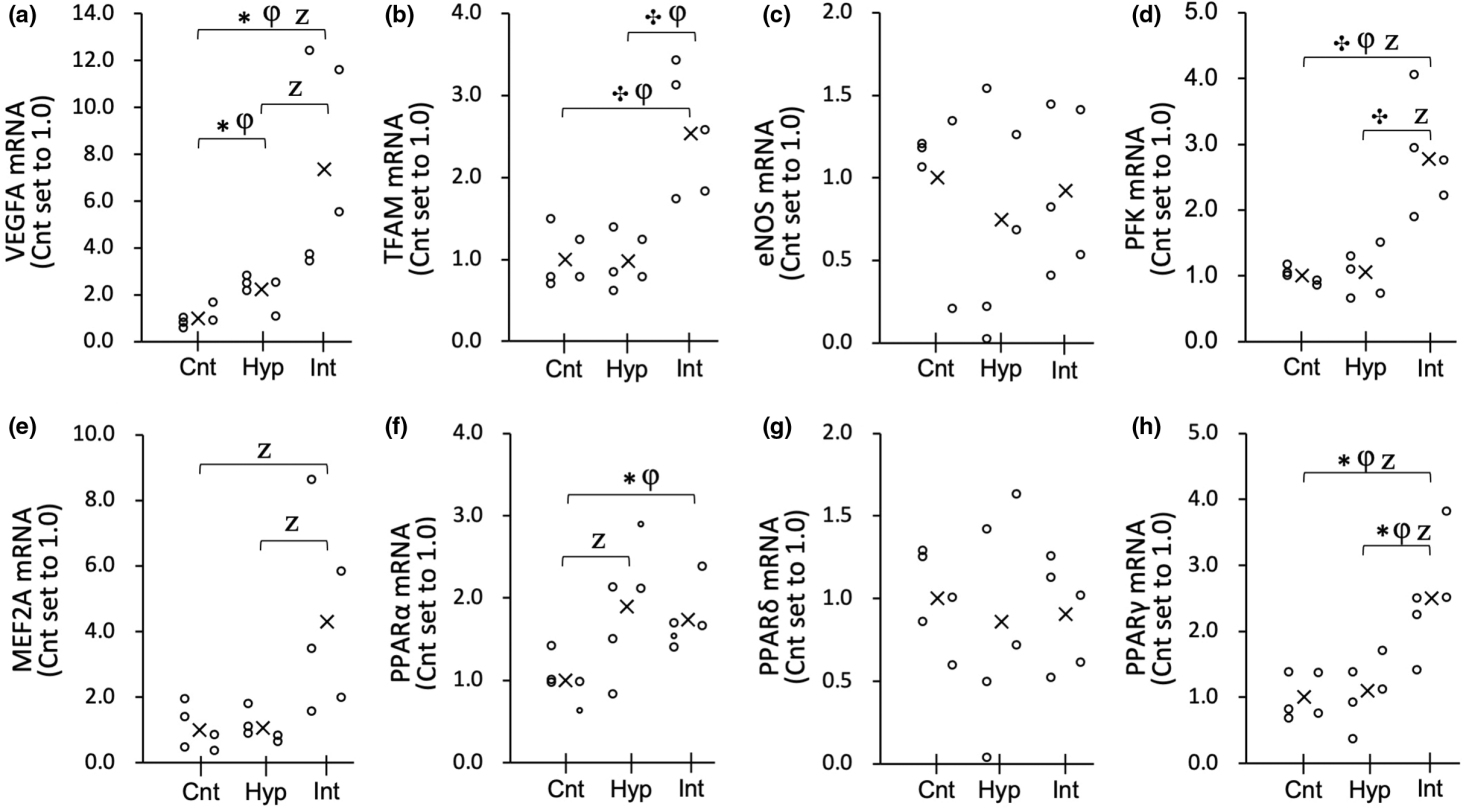
mRNA expression levels of VEGFA (a), TFAM (b), eNOS (c), PFK (d), MEF2A (e), PPARα (f), PPARδ (g), and PPARγ (h) in the red regions of the gastrocnemius muscle (Gr) at 3 h after acute hyperoxic interventions in the Experiment 1. Values are represented as individual data plot (circle) and mean value (x). The number of mice was 5 per group. Bayes factor: ✻, ≥ 3; ✣, ≥ 10; ✦, ≥30. φ, the 95% confidential interval did not contain the mean value of target group for comparison. Z, as a reference value, NHST‐ANOVA and Tukey's post hoc test, *p* < 0.05.

**FIGURE 3:**
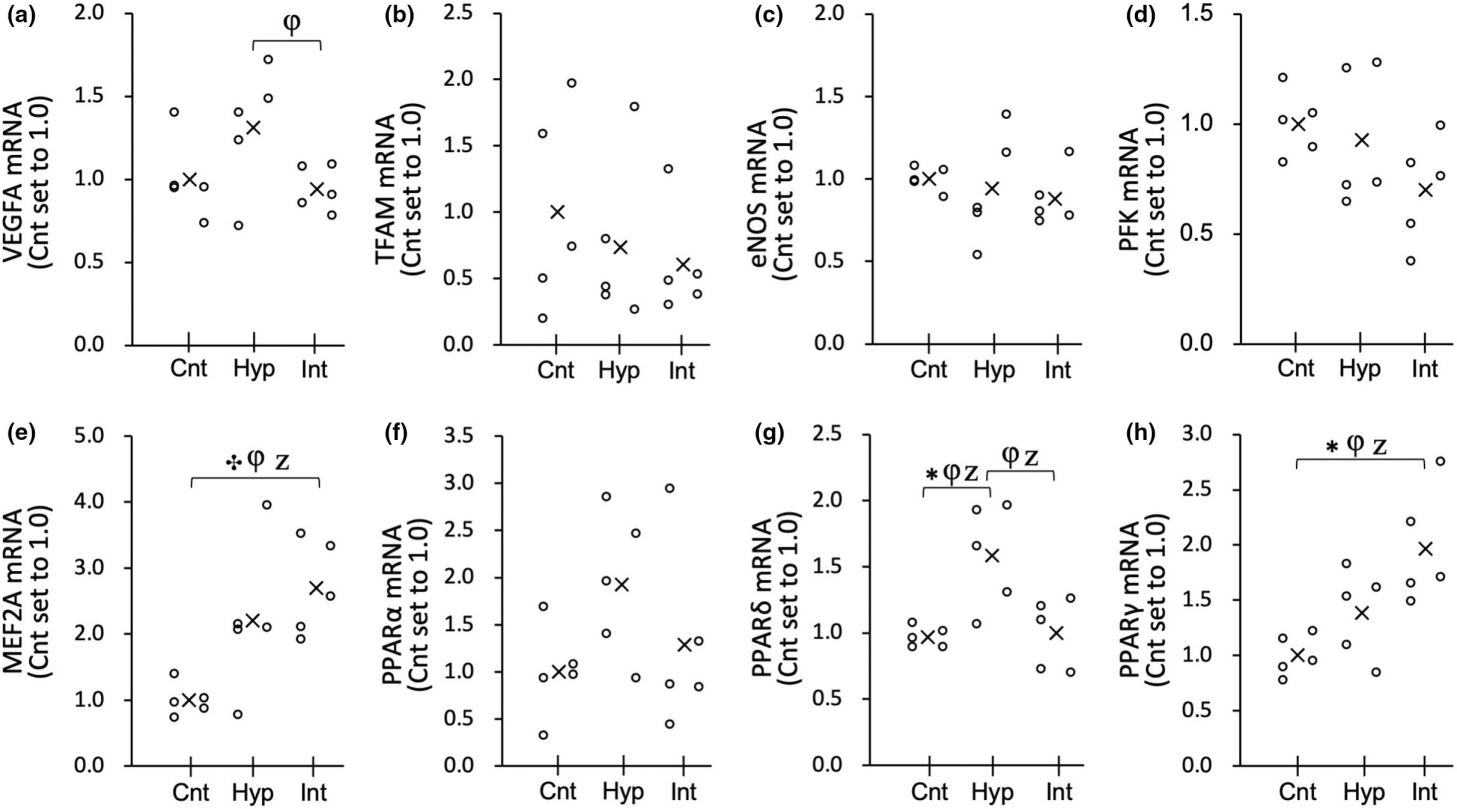
mRNA expression levels of VEGFA (a), TFAM (b), eNOS (c), PFK (d), MEF2A (e), PPARα (f), PPARδ (g), and PPARγ (h) in the white regions of the gastrocnemius muscle (Gw) at 3 h after acute hyperoxic interventions in the Experiment 1. Values are represented as individual data plot (circle) and mean value (x). The number of mice was 5 per group. Bayes factor: ✻, ≥ 3; ✣, ≥ 10; ✦, ≥ 30. φ, the 95% confidential interval did not contain the mean value of target group for comparison. Z, as a reference value, NHST‐ANOVA and Tukey's post hoc test, *p* < 0.05.

**FIGURE 4:**
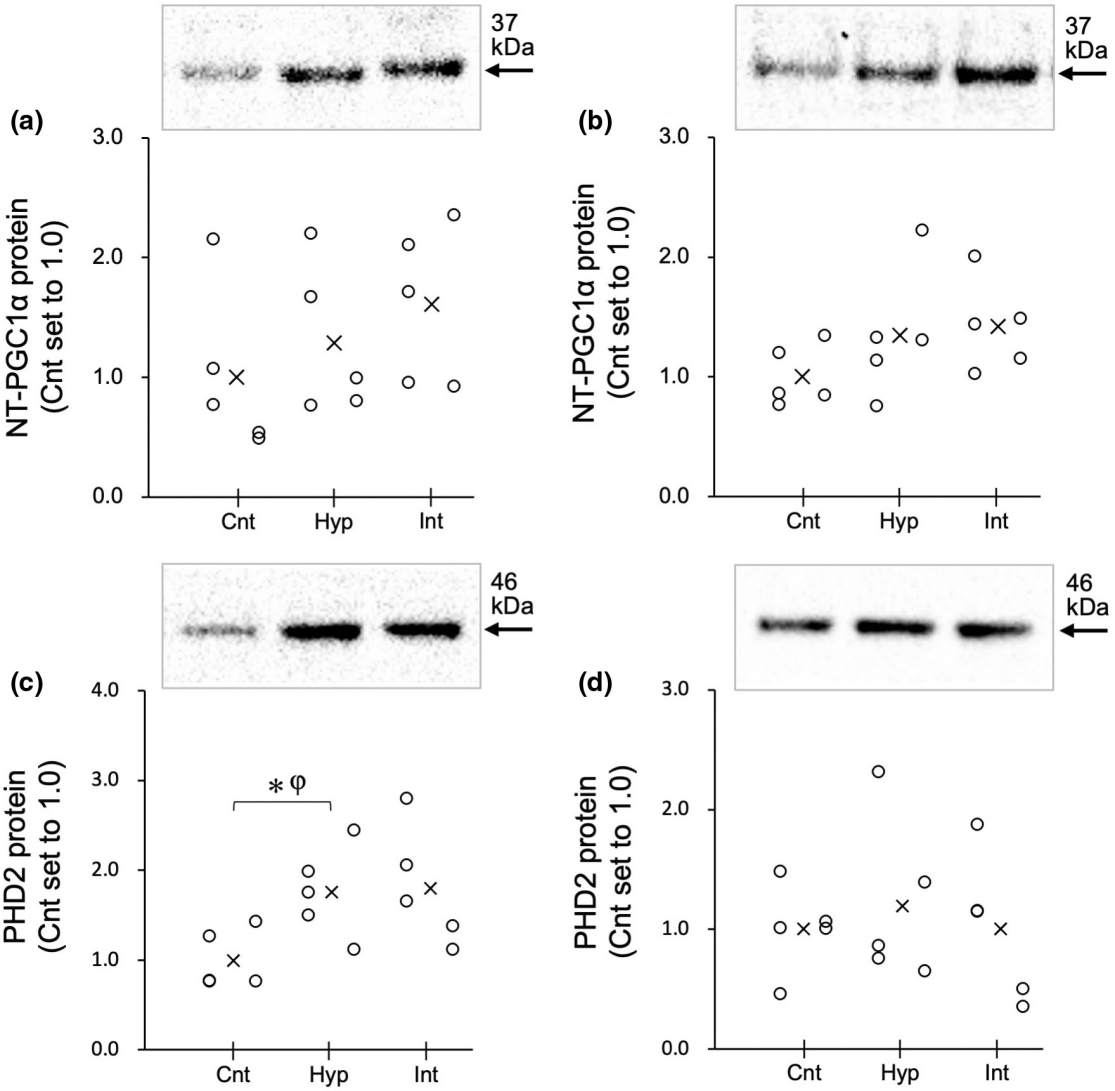
Nuclear protein levels for NT‐PGC1α (a, b) and PHD2 (c, d) in the red (a, c) and white (b, d) regions of the gastrocnemius muscle at 3 h after acute hyperoxic interventions in the Experiment 1. The densities of the specific bands were normalized to the densities of all protein bands in each lane on the membrane (Gilda & Gomes, 2013). This normalizing procedure was confirmed to be superior to using β‐actin as a loading control (Gilda & Gomes, 2013). Subsequently, the normalized densities of the bands were further normalized to the same sample that was run on every gel and transferred to every membrane, as reported by the author (Suzuki, 2021). Values are represented as individual data plot (circle) and mean value (x). The number of mice was 5 per group. Bayes factor: ✻, ≥3. φ, the 95% confidential interval did not contain the mean value of target group for comparison.

**FIGURE 5:**
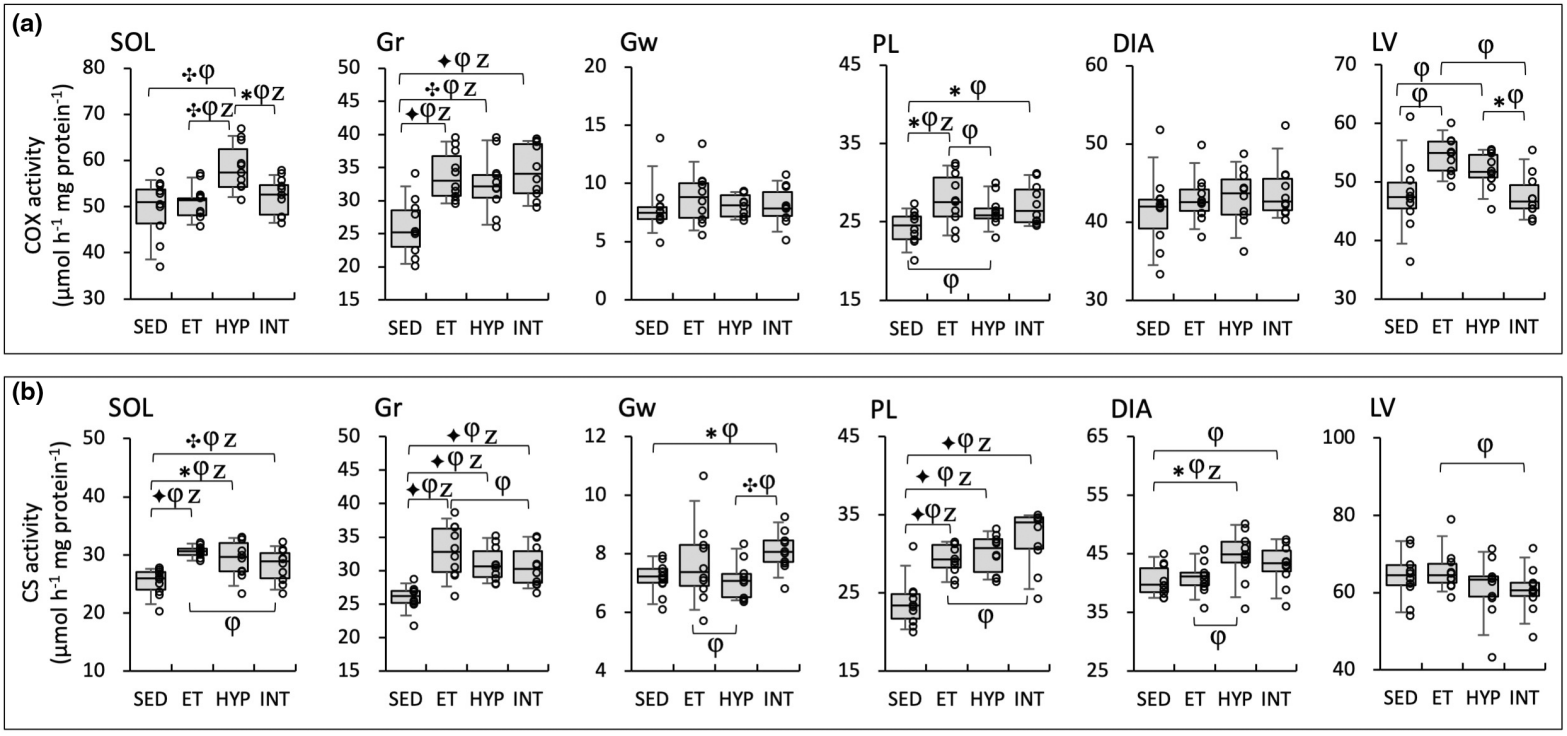
Enzyme activity values for COX (a) and CS (b) in the Experiment 2. Values are expressed as box and whisker plots with 5th, 25th, 50th, 75th, and 95th percentile. Dots are individual data points. Bayes factor: ✻, ≥ 3; ✣, ≥ 10; ✦, ≥ 30. φ, the 95% confidential interval did not contain the mean value of target group for comparison. Z, as a reference value, NHST‐ANOVA and Tukey's post hoc test, *p* < 0.05.

**FIGURE 6:**
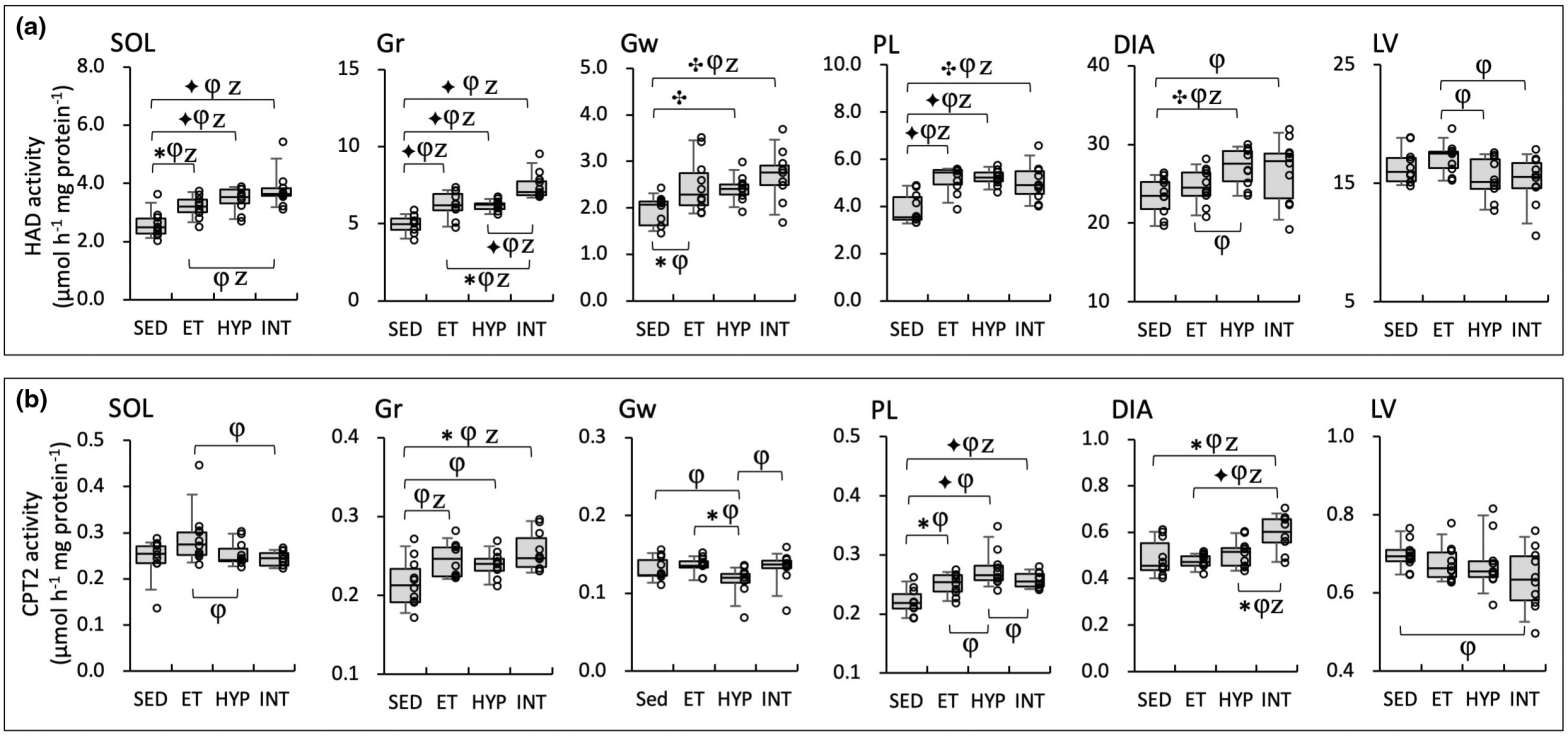
Enzyme activity values for HAD (a) and CPT2 (b) in the Experiment 2. Values are expressed as box and whisker plots with 5th, 25th, 50th, 75th, and 95th percentile. Dots are individual data points. Bayes factor: ✻, ≥ 3; ✣, ≥ 10; ✦, ≥ 30. φ, the 95% confidential interval did not contain the mean value of target group for comparison. Z, as a reference value, NHST‐ANOVA and Tukey's post hoc test, *p* < 0.05.

**FIGURE 7:**
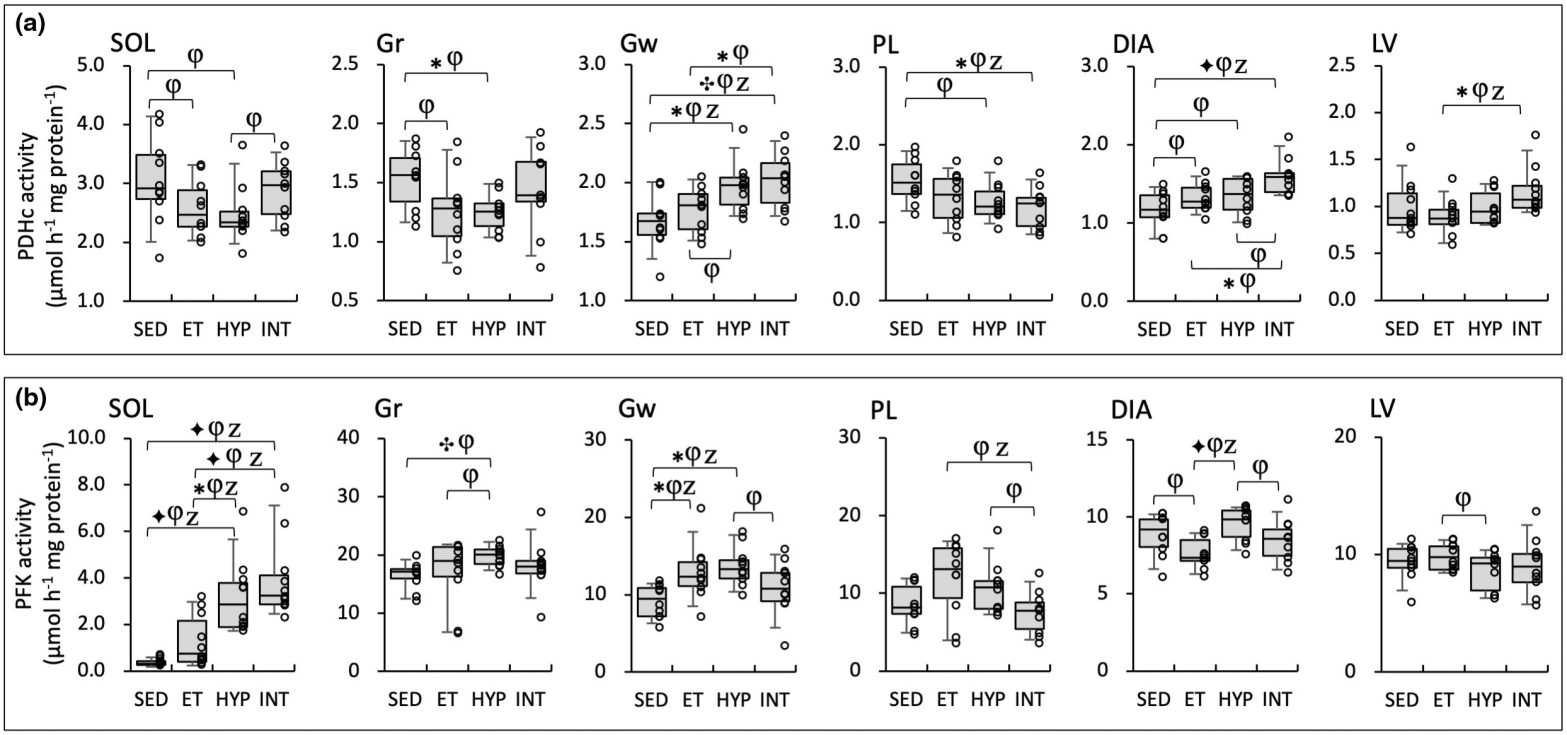
Enzyme activity values for PDHc (a) and PFK (b) in the Experiment 2. Values are expressed as box and whisker plots with 5th, 25th, 50th, 75th, and 95th percentile. Dots are individual data points. Bayes factor: ✻, ≥ 3; ✣, ≥10; ✦, ≥ 30. φ, the 95% confidential interval did not contain the mean value of target group for comparison. Z, as a reference value, NHST‐ANOVA and Tukey's post hoc test, *p* < 0.05.

**FIGURE 8:**
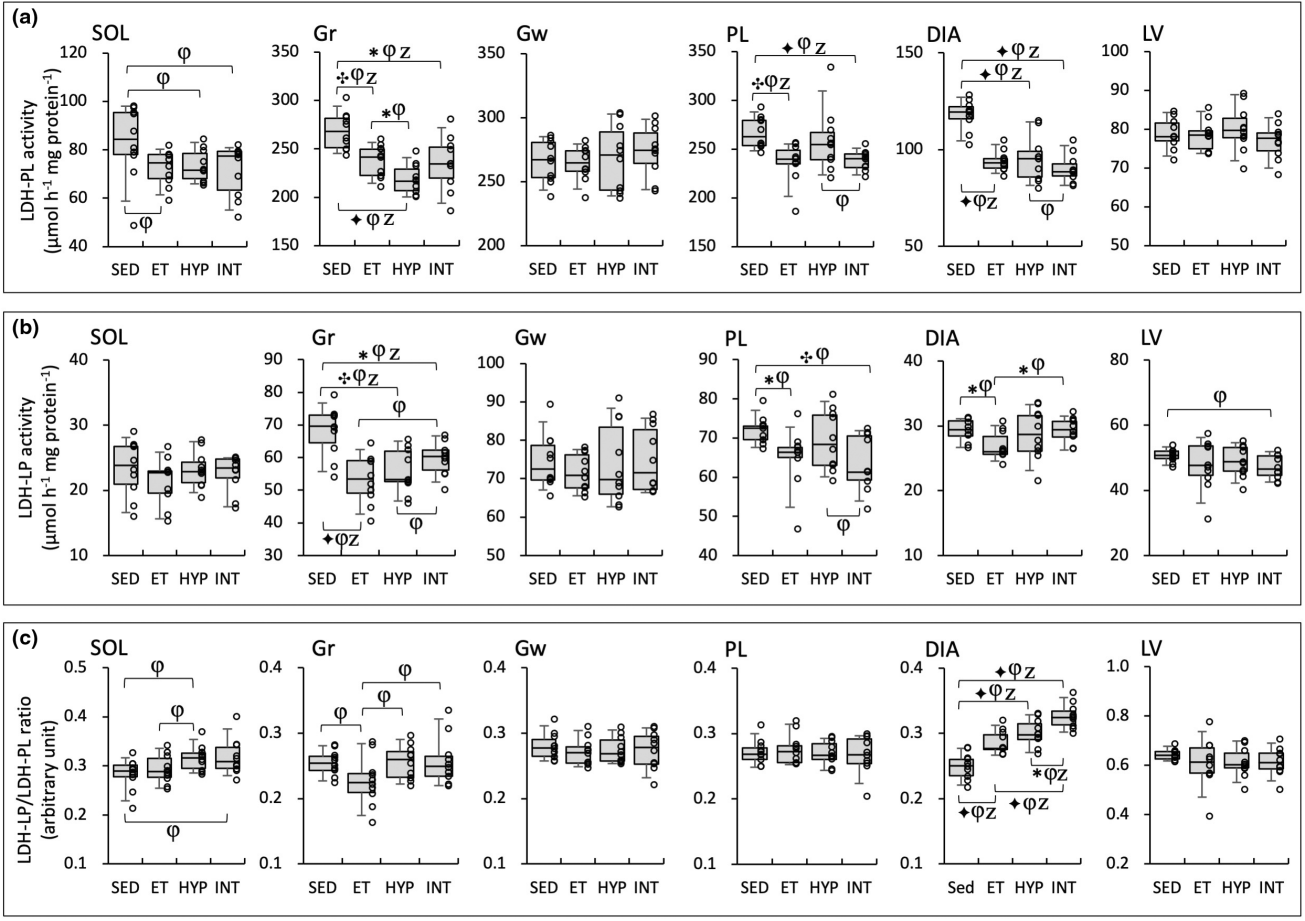
Enzyme activity values for LDH‐PL (a), LDH‐LP (b), and LDH‐LP/LDH‐PL ratio (c) in the Experiment 2. Values are expressed as box and whisker plots with 5th, 25th, 50th, 75th, and 95th percentile. Dots are individual data points. Bayes factor: ✻, ≥ 3; ✣, ≥10; ✦, ≥30. φ, the 95% confidential interval did not contain the mean value of target group for comparison. Z, as a reference value, NHST‐ANOVA and Tukey's post hoc test, *p* < 0.05.

**FIGURE 9:**
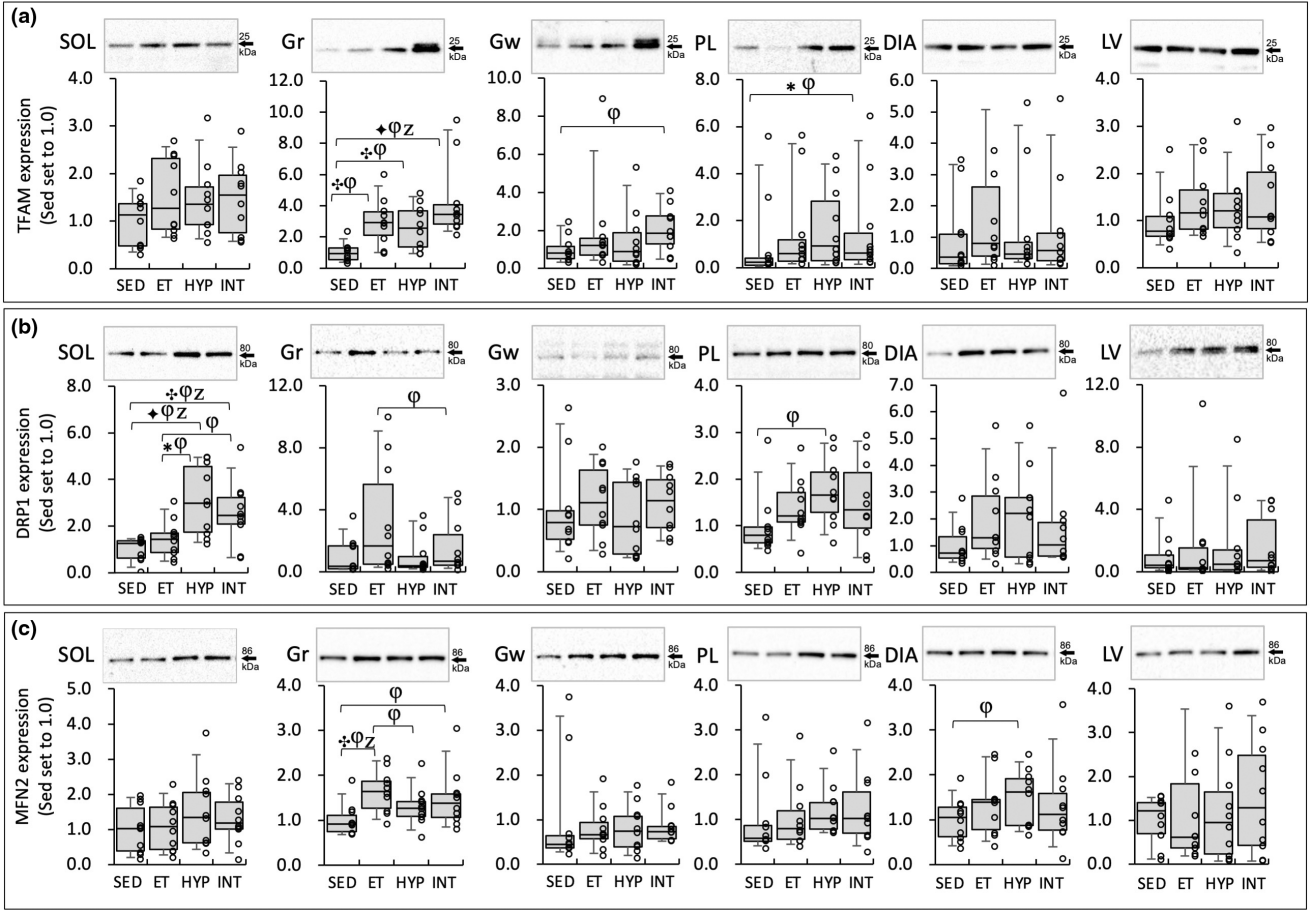
Protein levels for TFAM (a), DRP1 (b), and MFN2 (c) at 48 h after the last exercise session in Experiment 2. The densities of the specific bands were normalized to the densities of all protein bands in each lane on the membrane (Gilda & Gomes, 2013). This normalizing procedure was confirmed to be superior to using β‐actin as a loading control (Gilda & Gomes, 2013). Subsequently, the normalized densities of the bands were further normalized to the same sample that was run on every gel and transferred to every membrane, as reported by the author (Suzuki, 2021). Values are expressed as box and whisker plots with 5th, 25th, 50th, 75th, and 95th percentile. Dots are individual data points. Bayes factor: ✻, ≥3; ✣, ≥10; ✦, ≥ 30. φ, the 95% confidential interval did not contain the mean value of target group for comparison. Z, as a reference value, NHST‐ANOVA and Tukey's post hoc test, *p* < 0.05.

**FIGURE 10:**
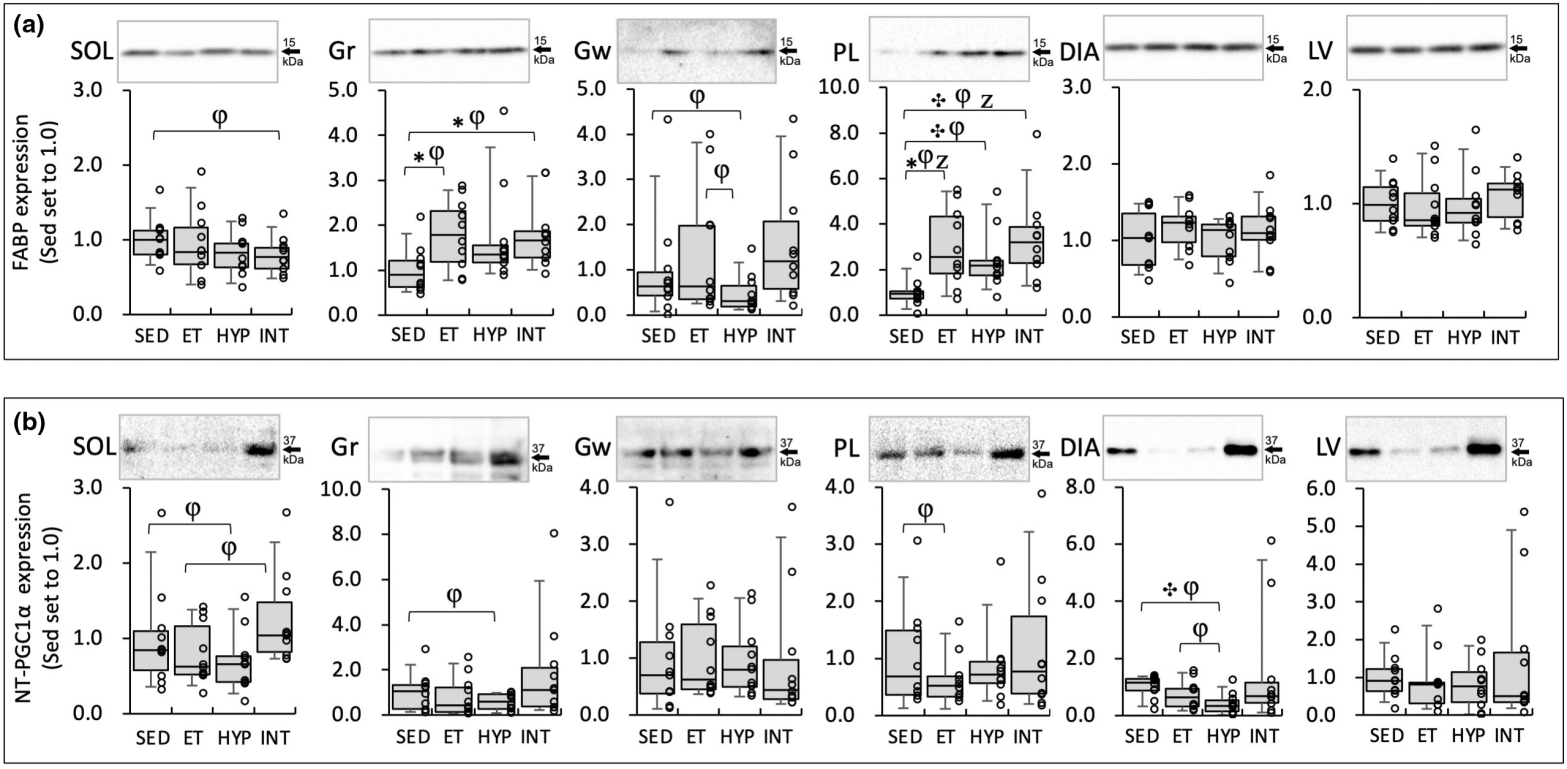
Protein levels for FABP (a) and nuclear NT‐PGC1α (b) at 48 h after the last exercise session in Experiment 2. The densities of the specific bands were normalized to the densities of all protein bands in each lane on the membrane (Gilda & Gomes, 2013). This normalizing procedure was confirmed to be superior to using β‐actin as a loading control (Gilda & Gomes, 2013). Subsequently, the normalized densities of the bands were further normalized to the same sample that was run on every gel and transferred to every membrane, as reported by the author (Suzuki, 2021, 21). Values are expressed as box and whisker plots with 5th, 25th, 50th, 75th, and 95th percentile. Dots are individual data points. Bayes factor: ✻, 3 ≤; ✣, 10 ≤; ✦, ≤ 30. φ, the 95% confidential interval did not contain the mean value of target group for comparison. Z, as a reference value, NHST‐ANOVA and Tukey's post hoc test, *p* < 0.05.

The statement on the *p*‐value by the American Statistical Association (Wasserstein & Lazar, 2016) highlighted the widespread misuse and misunderstandings surrounding *p* values derived from null hypothesis significance testing (NHST) and suggested alternative approaches such as Bayes factors. Numerous articles have pointed out the limitations of NHST testing (e.g., Amrhein & McShane, 2019; Hentschke & Stüttgen, 2011; Nakagawa & Cuthill, 2007; Smith, 2018). It has been reported that Bayesian hypothesis testing, specifically using Bayes factors in the public domain JASP program, is a replacement for NHST in most situations (Kelter, 2020). While NHST can only reject the null hypothesis, the Bayes factor can provide evidence for both the null and the alternative hypothesis, making confirmation of hypotheses possible (Kelter, 2020). Thus, this study used Bayesian data analysis for statistical significance testing. All statistical analyses were performed using the JASP. Differences between groups were examined using Bayesian ANOVA data analysis. If the Bayes factor (BF, BF_10_ of JASP) was greater than 3.0, the study confirmed the difference as statistically significant. When 95% confidence interval (CI) values did not include the mean value of target group for comparison, differences were considered to be biologically important (Du Prel et al., 2009; Gardner & Altman, 1986) and were described as a substantial change. In figures and tables, *p* values, obtained through NHST‐ANOVA and Tukey's post hoc test, were only noted as reference values. Bayesian correlation analysis was used to determine the correlations between two variables. The correlation was considered statistically significant when the BF was greater than 3.0 (Nuzzo, 2017). Data are presented as individual plots and mean value of each group (X) in Figures 2, 3, 4, both individual plots and box and whisker plots with 5th, 25th, 50th, 75th, and 95th percentiles in Figures 1, 5, 6, 7, 8, 9, 10. In tables, data are expressed as means ± standard deviation (SD) with the range in brackets.

## 3 RESULTS

### 3.1 Experiment 1: Acute response to hyperoxic exposure

At 3 h after acute hyperoxic exposure, VEGFA mRNA in Gr was significantly higher in the Int (7.3‐fold) and Hyp groups (2.2‐fold) than in the Cnt group (BF ≥3.0, Figure 2a). The mRNA levels of TFAM, PFK, and PPARγ (Figure 2b,d,h, respectively) showed significantly greater values in the Int group compared to the Cnt and Hyp groups (BF ≥3.0). PPARα mRNA levels were significantly greater in the Int group than in the Cnt group (BF ≥3.0, Figure 2f). eNOS and PPARδ mRNA levels were not affected by hyperoxic exposure (Figure 2c,g, respectively). For MEF2A mRNA levels, the Int group exhibited markedly higher values than the Cnt (4.3‐fold) and Hyp (4.1‐fold) groups, but the differences were not significant (Figure 2e). Nuclear NT‐PGC1α levels increased slightly in the Hyp (1.3‐fold) and Int (1.6‐fold) groups in Gr (Figure 4a) compared to the Cnt. In Gr, a significant positive correlation was observed between MEF2A mRNA levels and nuclear NT‐PGC1α protein levels (Table 2). MEF2A levels were also significantly correlated with TFAM mRNA levels (Table 2). Nuclear PHD2 protein expression levels in Gr were higher in both Hyp and Int groups (1.8‐fold) compared to the Cnt group; however, a significant difference was observed between the Hyp and Cnt groups (BF ≥3.0, Figure 4c).

**TABLE 2:** Correlations.

	Explanatory variable	Response variable	*r*	BF	*p*
Experiment 1					
Gr	MEF2A	NT‐PGC1α	0.547	4.79	0.035
	MEF2A	TFAM	0.860	≥30	<0.001
Gw	MEF2A	NT‐PGC1α	0.443	2.10	0.098
	MEF2A	TFAM	−0.072	0.390	0.80
Experiment 2					
SOL	HAD	Maximal work	0.377	3.32	0.04
Gr	HAD	Maximal work	0.433	6.91	0.017
	CPT2	Maximal work	0.395	4.11	0.031
	NT‐PGC1α	Maximal work	0.441	7.74	0.015
DIA	CPT2	Maximal work	0.453	9.24	0.012
	LDH‐LP/PL	Maximal work	0.433	6.83	0.017

*Note*: *r*, Pearson's product moment correlation coefficient. BF, Bayes factor. *p*, *p* value calculated by NHST.

In Gw, the mRNA levels of MEF2A (2.7‐fold) and PPARγ (2.0‐fold) showed significantly greater values in the Int group than in the Cnt group (Figure 3e,h, respectively). The mRNA levels of PPARδ in the Hyp group were significantly higher than those in the Cnt group (Figure 3g). MEF2 mRNA levels in Gw were significantly greater in the Int group than in the Cnt group (Figure 3e). mRNA levels of VEGF, TFAM, eNOS, PFK, and PPARα did not show notable changes. Protein levels of NT‐PGC1α and PHD2 were not remarkably altered in the Hyp and Int groups (Figure 4b,d, respectively).

For HIF1α target genes, VEGFA mRNA levels were upregulated after both continuous and intermittent hyperoxic exposure, but PFK mRNA levels were enhanced only after intermittent exposure in Gr. Nuclear PHD2 protein levels, a HIF1α target, increased similarly after both types of exposure, but a significant increase was observed after continuous exposure. Intermittent acute hyperoxic exposure had greater effects on gene expressions of PPARs. Moreover, intermittent hyperoxia enhances gene levels related to mitochondrial biogenesis (TFAM) in Gr.

### 3.2 Experiment 2: Chronic response of exercise training with intermittent hyperoxic intervention

#### 3.2.1 Body mass and maximal exercise capacity

After exercise training, the body weights of the HYP and INT groups were not significantly different from those of the ET group (Table 3). After 4 weeks of exercise training with hyperoxic exposure, body weight showed a significant increase in both the HYP and the INT groups. However, there was no significant increase in body weight observed in the ET group. Total work values were significantly elevated after 4 weeks of treadmill training in all three trained groups (BF ≥30, Figure 1b). Furthermore, total work values were significantly greater in the INT groups compared to the ET and HYP groups (BF ≥30). In essence, exercise training under short‐duration intermittent hyperoxia had additive effects on improving endurance exercise capacity.

**TABLE 3:** Changes in body mass values before and after exercise training.

	SED (*n* = 10)	ET (*n* = 10)	HYP (*n* = 10)	INT (*n* = 10)
Body mass (g)				
Pre‐ET	36.3 ± 1.68	36.5 ± 1.57	36.6 ± 1.05	36.7 ± 1.9
	[34.5−40.0]	[34.3−39.7]	[35.0−38.1]	[34.4−40.1]
Post‐ET	39.7 ± 1.39 (α)	37.5 ± 1.62 (ψ,φ)	37.9 ± 1.09 (α,ψ,φ)	38.7 ± 2.3 (α)
	[37.9−42.5]	[35.7−40.6]	[36.8−39.9]	[35.3−42.5]

*Note*: Values are presented as means ± SD with range in brackets.

ψ, the difference of mean values was significantly different from the SED group at BF ≥3.0. α, the difference of mean values was significantly different from pre‐ET values of each group at BF ≥3.0. φ, the 95% confidential interval did not contain the mean value of the SED.

#### 3.2.2 Enzyme activity

As for SOL, COX levels were significantly greater in the HYP group compared to the other three groups (BF ≥3.0, Figure 5a). In LV, COX values in the INT group were significantly lower compared to the HYP (BF = 4.0) group. In Gw and DIA, COX levels were not changed in the three trained groups. CS levels in Gw showed significantly greater values in the INT group compared to the SED (BF ≥3.0) and HYP (BF ≥10) groups (Figure 5b). Thus, COX levels in highly oxidative muscle were enhanced in the HYP group, while CS levels in glycolytic muscle were increased in the INT group.

HAD activity values in SOL, Gr, Gw, and PL were significantly higher in the three trained groups compared to the SED group (BF ≥3.0, Figure 6a). In Gr, HAD values in the INT group were significantly higher than those in the ET (BF ≥7.9) and HYP (BF ≥30) groups. Additionally, HAD values in SOL and Gr were significantly correlated with total work values (Table 2).

In DIA, CPT2 values were significantly higher in the INT group compared to the other three groups (BF ≥4.1, Figure 6b). CPT2 values in Gr and DIA showed a significant correlation with total work values (Table 2). In Gw, CPT2 levels were significantly lower in the HYP group than in the ET group (BF = 4.9). Thus, enzyme activity levels concerning fatty acid metabolism were enhanced in the INT group.

In Gw, PDHc activity levels were significantly higher in the INT group than in the ET group (BF = 3.4, Figure 7a). PDHc values were significantly higher in the INT group compared to the ET group (BF = 4.9) in DIA. Moreover, in LV, PDHc levels showed significantly greater values in the INT group compared to ET group (BF = 4.3). Thus, PDHc levels were enhanced in the INT group, thereby facilitating pyruvic acid utilization.

PFK activity levels in SOL were significantly higher in the HYP (BF = 7.2) and INT (BF ≥30) groups compared to the ET group (Figure 7b). PFK values were significantly higher in the HYP group compared to the ET group in DIA (BF ≥40). Activity levels of the rate‐limiting enzyme for glycolysis were increased in highly oxidative muscle in both HYP and INT groups. Meanwhile, in the respiratory muscle, the activity levels were enhanced only in the HYP group.

LDH‐PL activity levels in Gr were significantly lower in the HYP group than in the ET group (BF = 3.5, Figure 8a). LDH‐PL levels did not show notable changes in LV and Gw. In DIA, LDH‐LP levels were significantly greater in the INT group than in the ET group (BF = 3.9, Figure 8b). In SOL and Gw, LDH‐LP levels did not show notable changes.

In DIA, LDH‐LP/‐PL ratio values were significantly higher in the three trained groups (BF ≥30, Figure 8c). Furthermore, LDH‐LP/‐PL ratio levels were significantly higher in the INT group compared to the ET (BF ≥30) and HYP groups (BF = 5.0). A positive correlation was observed between LDH‐LP/‐PL ratio levels in DIA and total work values (Table 2). LDH‐LP/‐PL ratio values did not show remarkable changes in Gw, PL, and LV. Thus, lactic acid utilization was probably facilitated in the INT group.

#### 3.2.3 Protein levels

Protein levels were determined by analyzing tissue samples collected 48 h after the final performance test.

Protein levels of TFAM were significantly higher in the three training groups than in the SED group (BF ≥10, Figure 9a). TFAM expression levels were higher only in the INT group compared to the SED group, significantly in PL (BF = 6.7) and substantially in Gw (CI: 1.20–2.98). Notable changes in TFAM protein levels were not observed in the SOL, DIA, and LV. Thus, mitochondrial function was slightly facilitated in glycolytic muscle in the INT group.

DRP1 protein levels in SOL were significantly higher (BF = 6.9) in the HYP group and substantially higher (CI: 1.08–2.50) in the INT group compared to the ET group (Figure 9b). Notable changes in Drp1 levels were not observed in other tissues. MFN2 protein levels in Gr were significantly higher in ET group than in the SED group (BF ≥10, Figure 9c). Remarkable changes in MFN2 levels were not observed in other tissues. Thus, mitochondrial fission was probably facilitated in the HYP group.

In PL, FABP protein levels were significantly higher in all three trained groups compared to the SED group (BF ≥3.0, Figure 10a). In Gr, FABP levels were significantly higher in the ET (BF = 3.8) and INT (BF = 4.2) groups than in the SED group. Remarkable changes in FABP levels were not observed in other tissues. Thus, FABP expression levels were not affected by hyperoxic exposure.

In SOL, nuclear NT‐PGC1α protein levels were substantially higher in the INT group compared to the ET group (1.6‐fold, CI: 1.01–2.12, Figure 10b). In DIA, NT‐PGC1α levels were lower in the HYP group than in the SED group (BF = 13.6). In Gr, substantial decreases in NT‐PGC1α levels were observed in the HYP group than in the SED group (0.56‐fold, CI: 0.29–0.83). A significant positive correlation was observed between nuclear NT‐PGC1α levels in Gr and total work values (Table 2). Thus, a moderate increase in NT‐PGC1α levels probably contributes to improving muscle metabolic properties in the INT group.

## 4 DISCUSSION

### 4.1 Acute response to intermittent hyperoxic exposure

In the present study, in Gr, both continuous and intermittent hyperoxic exposure followed by 3 h normoxia increased the mRNA levels of VEGFA (Figure 2a). PFK mRNA levels were elevated after intermittent hyperoxia (Figure 2d). Therefore, it can be presumed that the cellular response to hypoxia, that is, HIF1α activation, identified as HIF‐target gene upregulation, was slightly greater in intermittent hyperoxia than in continuous one. Nuclear PHD2 protein levels in Gr were significantly increased after continuous hyperoxia compared to the Cnt group, but no significant difference was observed between the two types of exposure (Figure 4c). Thus, the subsequent degradation of HIF1α, identified as enhanced nuclear PHD2 protein levels, was slightly greater in continuous hyperoxic exposure rather than intermittent one. Taken together, HIF1α activation was modestly greater at 3 h after intermittent hyperoxic exposure rather than continuous one. Continuous hyperoxic exposure for 2 h followed by 4 h of normoxia was shown to upregulate HIF1α protein levels (Cimino et al., 2012). The present results found first to observe repeated short durations of hyperoxia to elicit HIF1α activation and subsequent degradation in the hind–leg muscle.

However, in Gw, the mRNA levels of VEGF and PFK, and PHD2 protein levels were not significantly changed after both types of exposure (Figures 3 and 4). In rats, resting blood flow in Gr was shown to be 6 times higher than that in the Gw (Armstrong & Laughlin, 1984). Moreover, capillary density values were approximately 2.8 times greater in the Gr than in the Gw (Suzuki, 2022). When inspired oxygen levels changed, tissue PO_2_ levels may change earlier in high blood flow and dense capillary regions, like Gr, than in regions with lower variables, like Gw. Thus, muscle tissues in Gr are likely to be more susceptible to changes in oxygen levels. In contrast, tissue oxygen levels in Gw may not change as quickly as blood oxygen levels. This idea presumably explains why the response to acute hyperoxia differs among muscle portions.

In this study, it was observed that acute intermittent hyperoxia led to an increase in the mRNA levels of PPARα in Gr and PPARγ in both Gr and Gw (Figures 2 and 3). However, acute continuous hyperoxia was found to significantly upregulate PPARδ in Gw. These findings imply that acute intermittent hyperoxic exposure has a greater effect on PPARs. PPARs are known to regulate fatty acid metabolism via upregulation of HAD and CPT2 expression levels.

Additionally, in Gr, the levels of nuclear NT‐PGC1α protein were positively correlated with mRNA levels of its co‐activator MEF2A (Table 3). These two variables in Gw showed a similar pattern after both continuous and intermittent hyperoxic exposure (Figures 3 and 4), but the correlation was not significant (Table 3). It has been shown that MEF2A interacts with PGC1α and regulates mitochondrial biogenesis (Ramachandran et al., 2008). PGC1α is known to regulate various transcription factors, including TFAM and nuclear respiratory factors (Wu et al., 1999). The mRNA levels of TFAM after acute intermittent hyperoxia were significantly enhanced in Gr (Figure 2b) but were insignificantly reduced in Gw (by 39%, Figure 3b). MEF2A mRNA levels were significantly correlated with TFAM mRNA levels in Gr but not in Gw (Table 2). Thus, intermittent hyperoxia, rather than continuous exposure, has the potential to improve muscle oxidative metabolism predominantly in oxidative muscle portions.

### 4.2 Effects of exercise training under continuous or intermittent hyperoxia

The body weight showed a significant increase after 4 weeks of exercise training with continuous and intermittent hyperoxia (Table 3). This suggests that exercise training conducted under continuous or intermittent hyperoxia did not appear to cause distress in mice.

The present study demonstrated that endurance exercise training combined with short‐duration intermittent hyperoxic exposure (referred to as INT training) had an additive effect on improving endurance exercise performance (Figure 1b). However, training with continuous hyperoxia (referred to as HYP training) did not enhance exercise performance to a greater extent than the ET group. Maximal work values were significantly correlated with HAD levels in SOL and Gr, CPT2 levels in Gr and DIA, LDH‐LP/PL levels in DIA, and NT‐PGC1α levels in Gr (Table 2). Therefore, it is likely that these variables primarily contribute to improving endurance performance after the INT training.

PGC1α is composed of three regions: the N‐terminal region, the middle region, and the C‐terminal region. The N‐terminal region plays a crucial role in controlling PGC1α's interaction with other transcription factors such as NRF1 (Wu et al., 1999), PPARγ (Puigserver et al., 1998), and MEF2C (Puigserver et al., 2001). Previous research has shown that overexpression of PGC‐1α enhances oxidative enzyme levels in rat skeletal muscles (Puigserver et al., 1998). In the present study, in SOL, nuclear NT‐PGC1α levels were substantially higher in the INT group (1.6‐fold) than in the ET group at 48 h after the last exercise (Figure 10b). However, NT‐PGC1α levels in Gr showed a significant positive correlation with maximal work values (Table 2). This notion indicates that NT‐PGC1α levels most likely contribute to the enhanced endurance performance observed after the INT training (Figure 1b).

Expression levels of HAD were shown to be regulated by PPARα (Iemitsu et al., 2002), and those of CPT2 are regulated by PPARα (Barrero et al., 2003) and PPARδ (Djouadi et al., 2005). The CPT complex plays a crucial role in facilitating the entry of long‐chain fatty acids from the cytosol into the mitochondrial matrix for beta‐oxidation. It is considered a key enzyme in fatty acid utilization. In this study, HAD activity levels in Gr were significantly higher in the INT training group compared to the ET group (Figure 6a). HAD values in SOL and Gr were positively correlated with total work values (Table 2). Additionally, CPT2 activity values were significantly increased after the INT training in DIA (Figure 6b). CPT2 values in Gr and DIA were also significantly correlated with total work values (Table 2). After the HYP training, however, the levels of HAD in DIA and CPT2 in PL were insignificantly higher compared to the ET group (Figure 6). This indicates that, in oxidative muscles, the INT training is more effective in promoting fatty acid metabolism than the HYP training. Exercise training under intermittent hypoxia (14% O_2_), in a previous study, did not show a further increase in HAD and CPT2 activity levels (Suzuki, 2022). Thus, the INT training used in this study has the potential to enhance fatty acid metabolism in the hind–leg muscles and the diaphragm.

PDHc is a complex of three enzymes that convert pyruvate into acetyl‐CoA, which is then used in the citric acid cycle. This complex serves as a link between the glycolysis metabolic pathway and the citric acid cycle. The current study discovered that the INT training significantly increased PDHc activity levels in Gw, DIA, and LV (Figure 7a). During cycling exercise, PDHc activation in the human vastus lateralis muscle was directly proportional to the relative aerobic power output (percent maximal oxygen consumption; Spriet & Heigenhauser, 2002). Therefore, the higher levels of PDHc activity observed after the INT training likely enable individuals to perform exercises at higher intensities. In a previous study conducted by the author, endurance training with intermittent hypoxia (14% O_2_) upregulated PDHc activity levels only in LV (Suzuki, 2022). As a result, the current INT training likely relies heavily on pyruvic acid utilization.

LDH enzyme exists in a tetramer formation, with five LDH isozymes composed of different ratios of the two subunits M and H (Markert, 1963), encoded by the LDHA and LDHB genes, respectively (Li, 1989). The H isomer is predominantly found in the myocardium and converts lactate to pyruvate in aerobic environments. The M isomer is abundant in skeletal muscles and converts pyruvate to lactate in anaerobic conditions. Unlike the LDHB gene, the LDHA gene possesses hypoxia recognition sites in its promoter sequence, making it responsive to HIF1α (Semenza et al., 1994). Consequently, the transcription of LDHA is upregulated by acute hypoxia (Firth et al., 1995), while LDHB generally shows no response to hypoxia (Osis et al., 2020). PGC1α was shown to regulate LDHB expression levels (Liang et al., 2016). Overexpression of LDHB in muscle has been shown to enhance endurance exercise performance and oxygen consumption during exercise in mice (Liang et al., 2016). In the present study, the LDH‐LP/‐PL ratio (Figure 8c) was observed, potentially indicating the H/M isomer ratio. Following INT training, marked increases in the LDH‐LP/‐PL ratio were observed in DIA. This suggests that the INT training may increase the expression of the LDH‐H isomer, facilitating the use of lactate as a fuel during exercise. In DIA, the activity levels of LDH‐LP/‐PL ratio values were found to have a positive correlation with total work values (Table 2). In addition, PDHc activity levels in DIA were significantly enhanced after the INT training (Figure 7a). These findings suggest that the INT training likely enhanced lactate oxidation during exercise, ultimately leading to an improvement in endurance capacity.

In the present study, it was found that HYP training did not lead to improvements in endurance performance to a greater extent than the ET group (Figure 1b). HYP training did result in significant enhancements in COX levels in SOL (Figure 5a). However, CPT2 levels were significantly decreased in Gw (Figure 6b). On the other hand, HYP training significantly enhanced PFK activity levels in SOL and DIA (Figure 7b). Thus, the present study found that HYP training significantly increased glycolytic enzyme levels in highly oxidative muscle and respiratory muscle. Additionally, HYP training slightly enhanced the index of lactate usage in highly oxidative muscle. However, it only had a modest effect on the enzyme levels related to oxidative metabolism in hind–leg muscle. As a result, HYP training did not lead to improved endurance exercise performance to a greater extent than the ET group, because endurance exercise is primarily dependent on oxidative metabolism.

## 5 CONCLUSION

This study is the first to demonstrate that the INT training, which involves exercise training with short‐duration intermittent hyperoxic intervention (30% O_2_) every other day for 4 weeks, has additional benefits in improving endurance performance. The INT training has been found to enhance enzyme activity levels associated with fatty acid metabolism in skeletal muscles. Moreover, it also promotes enzyme activity levels related to pyruvate metabolism in the hind–leg muscles, diaphragm, and heart. However, it should be noted that training under continuous hyperoxia (the HYP training) did not lead to improved endurance performance to a greater extent than the exercise training alone did, likely due to lesser improvements in oxidative enzyme activity levels. These findings underscore the potential advantages of the INT training as a strategy for enhancing endurance performance. To further investigate the significant effects of the INT training on endurance performance in athletes, additional experiments should be conducted using well‐trained animals.

## AUTHOR CONTRIBUTIONS

The author J.S. was involved in the conception and design of the study, analyzing the data, and preparing the first draft of the manuscript. The author revised the draft manuscript and approved the final concept. The author agrees to be accountable for all aspects of the work in ensuring questions relating to the accuracy and integrity of any part of the work are appropriately investigated and resolved.

## FUNDING INFORMATION

This work was supported by JSPS KAKENHI Grant Number 23K10579.

## CONFLICT OF INTEREST STATEMENT

None declared.

## ETHICS STATEMENT

All procedures were approved by the Animal Care and Use Committee of Hokkaido University of Education (No. 7, approved on 2023/4/1).

## PATIENT CONSENT STATEMENT

Not applicable.

## PERMISSION TO REPRODUCE MATERIAL FROM OTHER SOURCES

Not applicable.

## Data Availability

Data file that supports the present results is available at https://docs.google.com/spreadsheets/d/1qjlPaSGbsS8SRFcT6nKywXm7ltWtBY0‐/edit?usp=drive_link&ouid=103780764902759483879&rtpof=true&sd=true.
